# Bee Products in Dermatology and Skin Care

**DOI:** 10.3390/molecules25030556

**Published:** 2020-01-28

**Authors:** Anna Kurek-Górecka, Michał Górecki, Anna Rzepecka-Stojko, Radosław Balwierz, Jerzy Stojko

**Affiliations:** 1 Silesian Academy of Medical Sciences in Katowice, Mickiewicza 29, 40-085 Katowice, Poland; radoslaw.balwierz@gmail.com; 2 Department of Drug Technology, Faculty of Pharmaceutical Sciences in Sosnowiec, Medical University of Silesia, Jedności 8, 41-200 Sosnowiec, Poland; mgorecki@sum.edu.pl (M.G.); annastojko@sum.edu.pl (A.R.-S.); 3 Department of Toxycology and Bioanalysis, Faculty of Pharmaceutical Sciences in Sosnowiec, Medical University of Silesia, Ostrogórska 30, 41-200 Sosnowiec, Poland; jstojko@sum.edu.pl

**Keywords:** bee products, flavonoids, phenolic acids, skin care, therapeutic properties

## Abstract

Honey, propolis, bee pollen, bee bread, royal jelly, beeswax and bee venom are natural products which have been used in medicine since ancient times. Nowadays, studies indicate that natural bee products can be used for skin treatment and care. Biological properties of these products are related to flavonoids they contain like: chrysin, apigenin, kaempferol, quercetin, galangin, pinocembrin or naringenin. Several pharmacological activities of phenolic acids and flavonoids, and also 10-hydroxy-*trans*-2-decenoic acid, which is present in royal jelly, have been reported. Royal jelly has multitude of pharmacological activities: antibiotic, antiinflammatory, antiallergenic, tonic and antiaging. Honey, propolis and pollen are used to heal burn wounds, and they possess numerous functional properties such as: antibacterial, anti-inflammatory, antioxidant, disinfectant, antifungal and antiviral. Beeswax is used for production of cosmetics and ointments in pharmacy. Due to a large number of biological activities, bee products could be considered as important ingredients in medicines and cosmetics applied to skin.

Academic Editors: Raffaele Capasso and Lorenzo Di Cesare Mannelli

## 1. Introduction

Nowadays, alternative medicine, which employs natural biologically active substances obtained from bee products, is getting more and more attention. Bee products have been used not only in treatment, but also for skin care as ingredients of cosmetics. The effect of bee products on the skin has also been proved by numerous studies, and the use of honey, propolis, bee pollen and bee venom in wound healing highlights their curative value [1,2,3,4]. Each bee product possesses specific active substances which determine its use for various skin problems. Honey, propolis, bee pollen, bee bread, beeswax and bee venom are the bee products which are used for medicinal purposes and cosmetic production.

Honey is a natural product which is made by bees from nectar and honeydew. Honey is a supersaturated solution of carbohydrates with numerous properties and wide use. Propolis, also called bee glue, is a resinous substance collected by bees from buds of trees, shrubs, and green plants. Both, propolis and honey were used in antiquity for embalming bodies, whereas folk medicine used honey for wound healing and pain relief [5]. Bee pollen is collected from plants and transported to the hive in form of pollen loads. The formation of loads involves moisturizing pollen with nectar or honey. Pollen for winter supplies, which is deposited in the honeycomb cells, undergoes lactic fermentation and produces bee bread. Bee bread and bee pollen are bactericidal and bacteriostatic agents [6,7]. Beeswax is a substance produced by glands located in the bee abdomen. Wax obtained from honeycombs constitutes a valuable ingredient used in cosmetology and pharmacy. Bee venom also called apitoxin produced by honeybee. It consists a complex mixture of different peptides and mast cell degranulating peptide, which therapeutic and cosmetic properties are used in many areas [8].

## 2. Selected Compounds of Bee Products

The chemical composition of bee products is quite diversified, and depends on the botanical composition, geographical origin, time of collection and environmental conditions [9,10,11]. However, each product made by bees has a specified composition and content of biologically active substances, which give specific properties to each bee product. The chemical composition determines the curative and properties of these products.

Honey contains at least 181 ingredients [12]. Honey is a supersaturated carbohydrates solution containing mainly glucose and fructose [13]. Moreover, honey can have in its composition of sucrose, rhamnose, trehalose, nigerobiose, isomaltose, maltose, maltotetraose, maltotriose, maltulose, melezitose, melibiose, nigerose, palatinose, raffinose, and erlose [14,15]. It also contains enzymes, namely, glucose oxidase, amylase, catalase, peroxidase, invertase, and lysozyme. Glucose oxidase produces hydrogen peroxide which is one of responsible substances for the bactericidal activity of honey [16]. Honey contains also organic acids: gluconic acid, citric acid, malic acid, lactic acid, succinic acid, oxalic acid, tartaric acid, formic acid, acetic acid, benzoic acid, and pyromucic acid. The acids originate from bee bodies and enzymatic conversions which occur during honey production. The content of these acids is higher in mature honeys. Phenolic acids and flavonoids, which are responsible for many biological properties and have antioxidative activity, are also important ingredients of honey. The group of phenolic acids includes derivatives of hydroxycinnamic acid and hydroxybenzoic acid. The derivatives of hydroxycinnamic acid are *p*-coumaric acid, caffeic acid, ferulic acid, and sinapic acid. Whereas, the derivatives of hydroxybenzoic acid include *p*-hydroxybenzoic, vanillic, syringic, salicylic and gallic acids and ellagic acid as a dimer of gallic acid [15]. In honey, flavonoids are represented by naringenin, hesperetin, pinocembrin, chrysin, galangin, quercetin and kaempferol. However, a significant decrease in the concentration of galangin, kaempherol, and myricetin is observed after honey has been heated, while pasteurization causes a substantial decrease in myricetin concentration [17]. Honey contains also essential oils, whose composition includes terpenes (thymol, bisabolol, farnesol, and cineol). Other components of honey comprise water, amino acids and proteins. Proline (50–80%) dominates among amino acids, and its increased presence indicates honey maturity [14]. Vitamins constitute a small group of compounds present in honey, and they are mainly: thiamine, riboflavin, pyridoxine, *p*-aminobenzoic acid, folic acid, pantothenic acid, and vitamins A, C, E. Honey contains also minerals: phosphorus, potassium, calcium, magnesium, sulfur, iron, copper, manganese, and zinc. Although there is only a small amount of trace-elements in honey, they are highly bioavailable. It was reported that copper, calcium, zinc, iron, manganese and magnesium from honey are characterized a bioavailability of 80–90% [18].

In terms of chemical composition, propolis is a very diverse product. At present, at least 300 active compounds have been identified in it [19]. Phenolic acids (caffeic, ferulic, chlorogenic, *p*-coumaric), benzoic acid, cinnamic acid and flavonoids are the most important biologically active compounds. Among flavonoids, we can enumerate chrysin, luteolin, apigenin, galangin, kaempherol, quercetin, pinostrobin, pinocembrin, and terpene compounds, whose content is 0.5% (bisabolol), and alcohols (cetyl, myricyl, mannitol and inositol) [20,21,22]. Propolis contains also minerals (calcium, magnesium, manganese, zinc, copper, iron, cobalt and selenium), vitamins (B1, B2, B6, C and E) and enzymes (succinate dehydrogenase, glucose-6-phosphatase, adenosine triphosphatase, acid phosphatase) [22,23].

Bee pollen comprises at least 200 biologically active substances. Proteins constitute about 22.7% of bee pollen composition, including 10.4% essential amino acids: methionine, lysine, threonine, histidine, leucine, isoleucine, valine, phenylalanine, tryptophan. Digestible carbohydrates constitute 30.8%, while the percentage of reducing sugars is 25.7%. Among the fatty acids present in bee pollen, we can list acids such as gamma-linolenic acid, arachidonic acid, and linoleic acid (0.4%). Additionally, nucleic acids and nucleosides are valuable components of bee pollen [2,24]. It contains also vitamins (B1, B2, B3, B5, B6, C, H, E) and minerals (potassium, calcium, phosphorus, iron, zinc, copper, manganese) [9].

Protein content in bee bread is 12% lower than its content in bee pollen. The content of reducing sugars increases by 40–50%, whereas the content of lactic acid rises to 3.1%. Bee bread contains vitamin K and enzymes which cannot be found in bee pollen [25,26]. Bee bread is also a good source of phenolic components. Among bee bread from different parts of the Baltic Region the *p*-coumaric acid, ferulic acid, caffeic acid, kaempherol, isorhamnetin, naringenin and quercetin were identified [27].

Royal jelly contains peptides: jelleines I, II, III, IV, proteins, carbohydrates, lipids, vitamins and minerals [28]. Among proteins we can list royalisin and enzymes: amylase, invertase, catalase, acid phosphatase, and lysozyme. Proteins of royal jelly are rich in exogenous amino acids. The carbohydrates in royal jelly are mainly monosaccharides: fructose, glucose and oligosaccharides. Lipids play an important role in royal jelly composition [29]. 10-hydroxy-*trans*-2-decenoic acid, 3-hydroxydodecanoic acid, and 11-oxododecanoic acid can be included into the most valuable ones [28]. 10-hydroxy-*trans*-2-decenoic acid (10H2DA) is the main and specific lipid component of this product. 10H2DA is used as a marker to validate the quality of royal jelly [28,30]. Royal jelly contains also volatile compounds such as phenol, guaiacol and methyl salicylate. In royal jelly, there are also present trace amounts of such bio-elements as potassium, sodium, magnesium, phosphorus, sulfur, calcium, zinc, iron, and copper. Royal jelly contains mainly vitamins from group B: thiamine, riboflavin, pyridoxine, pantothenic acid, nicotinic acid and biotin and it is also contains phenolic compounds: ferulic acid, quercetin, kaempherol, galangin and fisetin, pinocembrin, naringin and hesperidin, apigenin, acacetin, and chrysin [31,32].

Esters of acids and fatty alcohols are main constituents of beeswax and subsequent components, in respect of amount, are free fatty acids [33]. Among the latter, 10-hydroxy-*trans*-2-decenoic acid (10H2DA) exhibits antibacterial effect, which is important. Beeswax is composed of hydrocarbons and free fatty alcohols [34,35]. Free fatty alcohols such as triacontanol, octacosanol, hexacosanol, and tetracosanol are antioxidative and anti-inflammatory. Other substances are triterpenes, β-carotene, volatile compounds and phenolic compounds. Among flavonoids, the main role is played by chrysin, which relieves inflammation, has antimicrobial and regenerative effects. Sterols have a regenerative effect, whereas an antiseptic effect is provided by three components: 10-hydroxy-*trans*-2-decenoic acid, chrysin, and squalene [34,36].

Bee venom contains different peptides including melittin, apamine, adolapin, sekapin, prokamin and mast cell degranulating peptide [37]. Peptides are main components of bee venom. Among peptides especially melittin plays important role in inducing reactions associated with bee stings. Melittin induces membrane permeabilization and lyses cells. It possesses also biologically active amines like histamine, epinephrine, dopamine, norepinephrine and enzymes like phospholipase A2, hyaluronidase, acid phosphomonoesterase, lysophospholipase. Bee venom has another components than peptides including lipids, carbohydrates and free amino acids [8,38,39].

## 3. Bee Products as Raw Material for Medicines and Cosmetics Production

Honey in cosmetics is named “Honey” or “Mel” according to the International Nomenclature of Cosmetic Ingredient (INCI), it is an emollient or humectant, and exhibits moisturizing properties. Some cosmetics contain derivatives of honey, defined in the INCI as “Mel Extract” with moisturizing properties, “Hydrogenated Honey” which is humectant, and antistatic “Hydroxypropyltrimonium Honey”. Hydroxypropyltrimonium honey is used in shampoos and hair conditioners. More often the concentration of honey in cosmetics is up to 10%. Higher concentrations (up to 70%) are obtained by dispersing in oils, gels or polymer entrapment [40].

Most frequently, propolis has a form of aqueous or ethanol extracts. According to the INCI nomenclature, in cosmetics we can find it under the following names: propolis and propolis extract. Ethanol extracts of propolis are most frequently used. To obtain them, propolis is extracted with 70% ethanol, and then the extract is concentrated in reduced pressure conditions [41]. An aqueous extract of propolis is used in antifungal cosmetics, while propolis dissolved in fats is used to produce lipsticks.

Royal jelly can most frequently be found in cosmetics in a lyophilized form, and the higher percentage content of lyophilized royal jelly is, the less viscous cream becomes. However, royal jelly content does not affect emulsion stability. Preparations with a higher content of royal jelly are well absorbed, and do not leave greasy film. Creams with royal jelly have moisturizing properties especially in concentration of 0.5% and 1% [42].

In cosmetic manufacturing, bee pollen is used in a form of aqueous, lyophilized and lipid extracts. Active substances can be extracted with water, propylene glycols, glycerin and oils. Bee pollen extacts are used in cosmetic in concentrations 0.5–5% [43]. In natural cosmetics, dried grains of bee pollen—micronized and added to cosmetics—are also used.

Beeswax is used in cosmetics after honey has been removed from honeycombs, wax has been melted, and impurities have been separated. To do this, various types of wax extractors are used: solar, electric or steam ones. Yellow wax (*Cera flava*) or white wax (*Cera alba*) is used to produce cosmetics [34].

According to INCI, bee venom or apitoxin are defined as bee venom powder. It is yellow light powder obtained by collecting a large amount of bee venom by electric stunning with using a bee venom collector without harming the honey bee. Then bee venom has to be purified under strict laboratory conditions. In next step purified bee venom is diluted in water, centrifuged, lyophilized and refrigerated for use as cosmetic ingredient [44]. It is used as a cosmetic ingredients which possesses antiaging, anti-inflammatory and antibacterial, antifungal and antiviral effects. Bee venom is used to produce antiphotoaging and anti-acne products [8,44]. Bee venom is used in treatment psoriasis, atopic dermatitis and alopecia [39].

## 4. The Effect of Bee Products on the Skin

### 4.1. Honey

Honey is used in medicine including due to its antimicrobial effect, which results from the following factors: hydrogen peroxide, high osmotic pressure, high acidity, the presence of phenolic acids, flavonoids and lysozyme [45]. Honey inhibits the growth of bacteria and fungi by reducing their development on the skin surface. Honey is particularly suitable as a dressing for wounds and burns, and has also been included in treatments against pityriasis, tinea, seborrhea, dandruff, diaper dermatitis, psoriasis, hemorrhoids, and anal fissure [40]. Pinocembrin and lysozyme are responsible for antifungal properties. Lysozyme inhibits growth of yeast-like fungi [46]. The effect of honey on healing postsurgical wounds was documented [1]. Among 52 patients incisions on skin were covered with honey dressing. The aesthetic outcome after third and six months was rated. The width of the scars was smaller in compare to conventional dressing. After 5-day application of honey dressing, an analgesic effect was obtained and wound healing was accelerated in women after plastic surgeries. Honey induced extracellular Ca^2+^ entry results in wound healing. It is similar to role plays by Ca^2+^ signaling in tissue regeneration [47]. Moreover honey regulates the process of epithelial mesenchymal transition (EMT) and it has a positive impact on wound healing. The effect on EMT depends on the floral and origin of the honey [48]. Honey is the apitherapeutic agent in topical wounds treatment due to killing bacteria, ability to bacterial biofilm penetration, lowing wounds pH, Reducing pain and inflammation, promoting fibroblast migration and keratinocyte closure, promoting collagen deposition so honey has a potential role in the area of tissue engineering and regeneration. Honey should be considered to incorporate it to the biomaterial tissue templates for tissue regeneration. Honey was used in electrospun templates, cryogels or hydrogels [49]. The main problem of use honey in tissue engineering are: cytotoxicity of high concentrations of honey, the lack of prolonged release rates of the honey over time. So future research should focus on these aspects. Among different types of honey, a strong antibacterial effect was observed in manuka honey which contains larger amount of methylglyoxal than European honeys [50]. The antibiotic activity of manuka honey is estimated by Unique Manuka Factor (UMF) and methylglyoxal (MGO) markers [46]. Due to an increased content of glucose oxidase, a higher level of hydrogen peroxide than in European honeys can be observed [51]. Hydrogen peroxide is responsible for produce free radicals, which cause oxidative damage to bacterial cell walls. The antimicrobial effect of honey from New Zealand is also evident in undiluted honeys and it is not abolished by catalases, which differentiates manuka honey from other types of honey. This type of honey is used in the treatment of various wounds, including burns. The inhibition value against *Staphylococcus aureus* FDA 209P of manuka honey in dilutions from 1:2 to 1:128 is determined in the range of 2.0–4.5 [50]. Manuka honey is used in medicine to heal burns, ulcers and wounds difficult to heal, and brings satisfactory results. Manuka honey also soothes gum inflammation, and inhibits the formation of dental plaque, fights thrush, and prevents periodontitis [52]. Another variety of honey with antibacterial activity is Revamil from The Netherlands. The antibiotic factor in Revamil is the peptide defensin-1 [46]. Bee defensin-1 permeabilizes bacteria and inhibits their RNA, DNA and protein synthesis [49]. However in other varieties of honey also the phenolic compounds are responsible for antibacterial effect.

Honey is a bee product with a high nutritional value and regenerative properties that is why it is used in skin care products. A high content of carbohydrates, the presence of fruit acids and trace elements are responsible for its nutritional and regenerative effects. Thanks to osmosis, microcirculation in the dermal tissue is stimulated, which results in its better nutrition and oxygenation. In this way, metabolic processes are also stimulated, which leads to eliminating harmful metabolites, and increasing regenerative processes. Additionally, honey has hygroscopic properties, absorbing metabolites, and causing detoxification of the dermal tissue. This results in an increase in the skin tension, improvement of its elasticity, revitalizing its color, and smoothing out wrinkles [52]. Fruit acids, as honey components, provide an exfoliating effect for dead skin cells. Honey can be used as peeling agent in a sugared form [53]. As a result, many valuable nutritional components, including vitamins, can diffuse through the skin more easily. Xerosis is relieved by fatty acids and mineral salts in honey. Honey soothes skin irritations, it is a good cosmetic for chapped lips, rough, cracked hands, and frost bites. Honey is used in balms and bath products because of its toning, relaxing, conditioning effects related to the high content of simple sugars, the presence of essential oils, and bioelements [53]. Due to the presence of flavonoids, honey can also play an important role in sun protection by preventing skin irritation [40].

### 4.2. Propolis

Propolis is widely used in medicine. Thanks to its antiseptic properties it is used in dermatology to treat staphylococcal, streptococcal and fungal infections. Purulent skin infections, hidradenitis, intertrigo, cheilosis, and thrush, among other things, are treated with propolis. As reported the Propol T, which is a propolis preparation, is highly effective in treatment of skin burns [54]. There are comparable therapeutic effects when propolis and sulfathiazole are used, however, bee glue is safer, and has fewer adverse effects. Propolis is not only antimicrobial and anti-inflammatory but also it increases cicatrization and reduces pain. Chrysin, which is a flavonoid, provides an analgesic effect. Propolis used to treat burn wounds in pigs increased fibrolast proliferation, activation and growth capacity. Propolis stimulates glycosaminoglycan accumulation what is needed for granulation, tissue growth and wound closure. Propolis as apitherapeutic agent is more effectively than silver sulfadiazine. Accumulation of collagen type I in matrix of an injury stimulates the repair process because collagen type I is indispensable for the keratinocyte migration and reepithelization. Moreover, propolis increased accumulation of collagen type III what accelerates healthy process. The usage of propolis ointment to treat burns as a topical apitherapeutic product could contribute to reepithelization [3]. Topically applied propolis decreased persistent inflammatory in diabetic wounds by normalizing neutrophil and neutrophil elastase. Caffeic acid is responsible for anti-inflammatory effect of propolis [55]. Genistein from propolis accelerated wound healing and stimulated wound angiogenesis in mice with diabetes type-1 [56]. Furthermore propolis may be effective in healing in different animal models including animals with burns and diabetic wounds [3,55,56]. Moreover propolis is highly effective in the treatment of *Acne vulgaris*. Researchers confirmed the limitation of occurrence of *Cutibacterium acnes,* i.e., a bacterium which plays a key role in acne vulgaris pathogenesis, after ethanol extract of propolis was applied to the skin [57]. The ethanol extract of propolis inhibits also *Staphylococcus epidermidis*. Propolis is used to manufacture cosmetics for the skin with acne, and to produce drugs against bacterial and fungal infections [58]. Propolis in the concentration of 5–20% has regenerative, repair effects and protects against external factors. It can be used to produce anti-bedsores preparations, since it firms the dermal tissue and protects it against pathogenic microbes [59]. Propolis protects also from ultraviolet radiation, since it can absorb UV light due to the presence of caffeic acid, coumaric acid, and ferulic acid. Propolis is a good additive to sun blockers (creams, lotions, sticks, and lipsticks) due to its properties of a natural filter, as well as antioxidative, anti-inflammatory and regenerative effects [60]. Other researchers showed that Romanian propolis had photoprotective effects against UVB after topical application to 30 Swiss mice [61]. Propolis is also used to produce protective lipsticks. It is regenerative and antiviral in cold sores caused by herpex simplex virus. Flavones and flavonols from propolis, especially galangin, kaempferol, quercetin, have a high antiviral activity against herpes simplex virus type 1 in vitro [62]. Nolkemper et al. observed that both, aqueous and ethanol extracts of propolis were strongly antiviral against herpes simplex type 2 (HSV-2) [63]. Skin care with products based on propolis is helpful against fungal problems of the skin due to the presence of flavonoids (pinocembrin and pinobanksin), phenolic acids (caffeic acid) and terpenes [59]. Pinocembrin isolated from propolis inhibits the mycelial growth of *Penicillium italicum* by interfering energy homeostasis and cell membrane damage of the pathogen [64]. Shampoos with bee glue can be a natural alternative in treatment of dandruff and prevention of its recurrence due to its antifungal and anti-seborrheic properties. Propolis has also been used for manufacturing toothpastes. Bee glue inhibits the formation of dental plaque and is antimicrobial, thereby it reduces dental caries development. Propolis ethanol extracts inhibit the growth of cariogenic bacteria, which include mainly *Staphylococcus mutant* and *Staphylococcus sobrinus*. Glucosyltransferase makes bacteria produce glucan they feed on, which is insoluble in water. Propolis eliminates cariogenic bacteria, inhibits the activity of glucosyltransferase, and reduces adherent abilities of bacteria [65]. The conducted studies showed that the use of toothpaste with propolis reduced dental plaque by 34.3% annually, whereas normal paste reduced the plaque by 31.9%. After two-year use of the paste with propolis a further reduction of plaque by 12.4% was observed, while normal paste managed to reduce it only by 5%. Rinsing the mouth with water with 0.5% propolis content complements the oral cavity care. After 21 days, this solution was able to reduce dental plaque by 18.1% [66]. Propolis smoothes out wrinkles and has antiaging properties. A huge role is played here by antioxidants such as phenolic compounds and flavonoids which neutralize an unfavorable effect of free radicals on the skin. Bee glue lightens and smoothens the skin, reduces signs of fatigue and moisturizes it [59].

### 4.3. Royal Jelly

Royal jelly has a broad spectrum of biological activities which determine the effect of royal jelly on the skin, namely, antibacterial, anti-inflammatory, immunomodulatory, anti-allergic, antioxidant, toning, moisturizing, and antiaging [67]. Royal jelly is a bee product with strong antimicrobial activity within skin tissue, which is already evident in 20% concentration. Due to its anti-inflammatory activity, royal jelly relieves periodontal diseases, inflammation of the oral cavity, tongue and throat. Anti-inflammatory activity and wound healing results from its ability to inhibit the production of pro-inflammatory cytokines (TNF-α, IL-6, IL-1). Royal jelly has a protective effect on blood vessels and relieves hemorrhoids, and varicose veins of the lower extremities. It is used to treat lichen, ulcers, burns, bed sores, shingles, in all cases where the regeneration of epidermis is expected, wound epithelialization, nutritional effect, healing and antimicrobial activity. The effect of 5% royal jelly on ulcers on the diabetic foot has been studied. The treatment lasted 3 months and involved dressing the wound with 5% sterile royal jelly 3 times a week. Among eight treated ulcers, seven were cured, and in one case an improvement was observed [68]. Royal jelly promotes wound reepithelization. The keratinocytes are responsible for the elevated production of MMP-9 (matrix metalloproteinase-9) after incubation with a water extract of royal jelly. After applying water extract of royal jelly increased keratinocyte migration and wound closure rates. The component of royal jelly responsible for stimulating MMP-9 production is defensin-1. Moreover defensin-1 promotes reepithelization and wound closure. Similarly as in honey, defensin-1 is responsible for cutaneous wound closure by enhancing keratinocyte and MMP-9 secretion [69]. Royal jelly is effective in the treatment of wounds, and is successfully used in cosmetics for problem skin care. Royal jelly is an ingredient of preparations normalizing sebum secretion, for seborrheic skin, acne-prone skin where frequently skin lesions and small wounds occur [31]. Due to stimulating metabolism in tissues, royal jelly improves regenerative processes of tissues. Regenerative, nutritional and healing properties are used in balms, creams, and lotions. Immunomodulatory and antiallergenic activities of royal jelly are related to the properties of fatty acids, isolated from it. Both, 10HDA and 3-10-dihydroxydecanoic acid modulate immune response and lower the concentration of IL-2 and IL-10. Anti-inflammatory and immunomodulatory activities of royal jelly were used to treat atopic dermatitis, hypertrophy, hyperkeratosis and epidermis and dermis inflammation, possibly through a blend of TNF-specific low adjustment of IFN-gamma specific production and high adjustment of nitric-oxide synthase (NOS) expression [70]. 10-hydroxy-*trans*-2-decenoic acid, which is present in royal jelly, stimulates fibroblast production of collagen by inducting the production of transforming growth factor. As a result, royal jelly affects the production of collagen, which is an important factor that supports the skin [28]. Royal jelly is highly moisturizing, and affects hydration of the stratum corneum by retaining water in it. In consequence, the skin become more elastic and better moisturized [42].

### 4.4. Bee Pollen

Bee pollen, another bee product, can also affect the skin. Bee pollen is a potent antifungal, antimicrobial, antiviral, anti-inflammatory, immunostimulating agent, and it also facilitates the granulation process of burn healing [71]. Pollen ethanol extract is antimicrobial against *Staphylococcus aureus, Escherichia coli, Klebsiella pneumoniae, Pseudomonas aeruginosa,* and has an antifungal activity against *Candida albicans.* Flavonoids and phenolic acids provide antifungal and antibacterial properties of bee pollen. Anti-inflammatory activity of bee pollen is due to inhibiting the activity of enzymes participating in the development of inflammation, i.e., cyclooxygenase II and lipoxygenase. Phenolic acids, fatty acids and phytosterols are responsible for anti-inflammatory characteristics. Additionally, kaempferol inhibits hyaluronidase and elastase, which suppresses inflammatory response. Besides, topical application of ointment with pollen extract to treat burns has been studied, since bee pollen can regenerate damaged tissues [2].

Bee pollen is an active ingredient in cosmetics, usually in the concentration of 0.5–5% [43]. Its significant effect on the skin tissue is due to a high content of flavonoids. Their presence allows bee pollen to strengthen and seal capillaries, which is also increased by high vitamin C content, and that is why bee pollen is used in creams for couperose skin. Bee pollen affects cell metabolism, boosts regeneration and stimulates mitotic division. Bee pollen is used to produce shampoos and conditioners. Its sebo-balancing activity, which involves reducing sebum secretion, is used in preparations for oily hair. Bee pollen normalizes the activity of sebaceous glands due to presence of zinc, methionine and phospholipids. Moreover, sulphur containing amino acids, mainly cysteine, present in bee pollen strengthen hair shaft. Bee pollen is also added to anti-dandruff shampoos, since it limits fungal growth and stops itching of the scalp, but it still has moisturizing, conditioning and regenerating properties. Other researchers inform that a good solution would be to mix ethyl esters of essential unsaturated fatty acids from flaxseeds with bee pollen. Essential fatty acids (EFA) would play the role of lipid fraction solvent. Preparations with omega-3 and omega-6 acids enriched with diverse properties of bee pollen could help in the care of atopic skin, sensitive skin, and the skin more vulnerable to scarring [43].

### 4.5. Beeswax

When compared to other bee products, beeswax has the smallest range of biological activities. Kędzia [34] wrote that beeswax was added to ointments, liniments and creams used in treatment of various dermatoses, e.g., boils, wounds, atopic dermatitis, psoriasis, diaper dermatitis caused by *Candida albicans*. Beeswax is mainly used as an emulsifying agent. In cosmetics, beeswax is used as a stiffener, a substance providing elasticity, plasticity and increasing skin adhesiveness. Beeswax is the base for lipsticks, sticks and creams [72]. Beeswax has lubricating, softening activities and reduces transepidermal water loss from skin. Sterols, which are also components of intercellular space, provide these characteristics of beeswax. Squalene, 10-hydroxy-*trans*-2-decenoic acid and flavonoids (chrysin) provide antiseptic properties to this product, and protect the skin against pathogenic microorganisms. Beeswax constitutes a protective barrier against many external factors by forming a film on the skin surface. β-carotene present in beeswax is a valuable source of vitamin A, into which it is converted. Vitamin A delays collagen degradation, stimulates mitotic division in the epidermis, thus leads to sooner regeneration of the skin after damage [34,36].

The main effects of flavonoids and phenolic acids present in above bee products on the skin are presented in Table 1.

### 4.6. Bee Venom

Bee venom has been used in medicine in treatment but also as a cosmetic ingredient. Bee venom has a wide spectrum of biological activity. It exhibit antibacterial and anti-inflammatory effects so it can be used as a ingredient of anti-acne products. Bee venom shows inhibitory effects on *Cutibacterium acnes*. *Cutibacterium acnes* is the main factor inducing the inflammation in acne. An et al. [81] showed that topical application bee venom on mice skin, which previous obtained intradermally injected *Cutibacterium acnes* into ears, limited number of inflammatory cells and also reduced level of tumor necrosis factor (TNF)-α and interleukin IL-1β. Moreover, bee venom inhibited Toll like receptor (TLR2) and CD14 expression in tissue which has been injected *C. acnes*. These results indicate that bee venom can be used as anti-acne agent. Another researchers [82] also showed positive effects of cosmetics containing bee venom on acne vulgaris. Purified bee venom reduced number of *C. acnes* at concentration of 0.5 mg. Bee venom possesses bactericidal and bacteriostatic effects thanks to melittin [38]. It has a significant antibacterial effect against *Staphylococcus aureus*, *Staphylococcus epidermidis* and *Staphylococcus pyrogenes* [39,83]. Melittin is a toxic peptide that causes destruction of the bacterial cell wall [38]. Bee venom can be used in fungi and viral skin infections. The antifungal effect of bee venom against *Trichophyton mentagrophytes*, *Trichophyton rubrum*, *Candida albicans* and *Malassezia furfur* was proved [84,85,86]. Antiviral effect of bee venom on herpes simplex virus has been studied. Bee venom suppressed the replication this virus [87]. Moreover bee venom is a potential inhibitor of 5 α-reductase, which is responsible for converse testosterone into dihydrotestosterone and plays important role as hair growth promoter, what was confirmed in study on alopecia. Bee venom in different concentrations 0.001%, 0.005% and 0.01% was applied in compare 2% minoxidil. Researchers showed that bee venom promoted hair growth and inhibited transition from the anagen to catagen phase. Additionally bee venom inhibited the expression of SRD5A2 which encodes a 5-α-reductase [88]. Bee venom can play role as a new therapy in localized plaque psoriasis. Intradermal bee venom and intradermal bee venom combined with oral propolis constitute effective treatment of localized plaque psoriasis. Bee venom reduces level of IL-1β, TNF-α, and IL-6. Bee venom contains melittin, which blocks the expression of inflammatory genes. Additionally bee venom inhibits the COX-2 expression, so decrease production of prostaglandins which take part in inflammatory process [89]. Bee venom compounds possess various, sometime opposing immune-related effects. Some components of bee venom like apamin, histamine, mast cell degranulating (MCD) peptide and phospholipase A2 (PLA2) increase inflammatory response, while polypeptide adolapin inhibits prostaglandins synthesis and inhibit the activity of bee venom PLA2 and human lipoxygenase [90]. Anti-inflammatory effect of bee venom is used also in treatment atopic dermatitis. Patients who applied emollient with bee venom had lower eczema area, severity index and visual analogue scale value than patients who applied emollient without bee venom [84]. The biological activities of bee venom have been used in wounds healing. The mechanism of wound healing is associated with expressions of TGF-β1, fibronectin, vascular endothelial growth factor (VEGF) and collagen-I. The research, which was conducted in mice showed decreasing of wound size and increasing epithelial proliferation. Topical use of bee venom is effective especially in reducing size of wounds in animal model [83]. The bee venom is using in wound dressing combined with polyvinyl alcohol and chitosan. 4% bee venom in wound dressing in diabetic rats accelerated healing and limited inflammatory process [91]. Another study showed that 6% bee venom with chitosan supported wound healing [92]. Researchers indicated that bee venom stimulated human epidermal keratinocyte proliferation and migration. Bee venom joined with hydrogel increased collagen formation. Bee venom supports wound healing due to its anti-inflammatory, anti-microbial and also antioxidant activity. Effective action of bee venom is very important in human melanoma A2058 cells. Tu et al. exhibited that bee venom leads to apoptosis cell death by induction hydroxyl radicals [93]. Recently bee venom also has been used as antiwrinkle agent. As a cosmetic ingredient bee venom serum at a concentration of 0.006% was applied at amount 4 mL twice a day for 12 weeks among twenty-two women from South Korea. It caused decreasing total wrinkle area, total wrinkle count and wrinkle depth. Moreover bee venom possesses antimelanogenic activity by inhibiting tyrosinase-related proteins [94]. The study conducted by Han et al. [44] reported that bee venom exhibits photoprotective activity by reducting of the protein levels of matrix metalloproteinases. Bee venom effectively inhibits photoaging processes so it can be used for photodamaged skin. Gel containing 0.06% bee venom did not lead to photosensitive dermatitis what has been confirmed on animal model [8,44].

## 5. Allergic Adverse Effects of Bee Products

The use of bee products for cosmetic as well as medicine production can involve the occurrence of allergic reactions. An allergy to honey is seldom, and the most frequent allergen from honey that causes hypersensitivity reactions is bee pollen. Additionally, bee protein in honey can cause an allergy. Honey used to treat dermatoses undergoes thorough filtration to eliminate particles of bee pollen, which are the main cause of honey allergic reactions. Honey allergy is very rare but sometimes causes IgE-mediated hypersensitivity reaction [95]. In 2010 Basista conducted studies on beekeepers. None of them was hypersensitive to honey [96]. More than 26 allergenic substances were determined in propolis composition. Most frequently, an allergic reaction is caused by esters of caffeic acid and cinnamic acid derived from poplar buds. In hypersensitive people, they cause a contact allergic reaction. Due to the presence of these esters in other materials, cross allergic reaction can occur. The most potent alergens are: LB-1, i.e., the compound consisting of 3-methyl-2-butyl-caffeate (54.2%), 3-methyl-3-butyl-caffeate (28.3%), 2-methyl-2-butylcaffeate (4.3%), caffeic acid (1.3%), benzyl caffeate (1.0%), caffeic acid phenethyl ester (CAPE, 7.9%) and benzyl salicylate. An allergy to propolis is rare, and an allergic response was more frequently reported after topical application than an oral one. In the years 1989–2006, the World Health Organization registered only 26 notifications about side effects after the contact with bee glue, of which just six were considered certain, and the remaining ones were not fully credible. In healthy individuals, an allergy to propolis is rarely observed (0.64–1.3%), however, it occurs more frequently in people treated for allergies (1.2–6.7%). This hypersensitivity is manifested by atopic eczema after the application of ethanol extract of propolis [97]. Moreover, topical application of royal jelly in the form of ointments can cause skin rashes and eczemas [67]. Allergic and irritation reaction of bee venom have been associated with presence components likes: phospholipase A2, melittin, hyaluronidase. Phospholipase A2 is a major allergen which is responsible for inducing immuno-globulin E (IgE) [98]. Melittin causes cell lysis and fusion in addition to activation of phospholipase A2. Hyaluronidase is a next allergen in venom, which is responsible for changes in cell membranes. It caused spread of venom toxin through the gaps between cells. However, bee venom can be toxic when large amount of venom is inoculated into body [98]. However, Han et al. indicates that long term topically treatment with bee venom is safe what confimed their study [94].

## 6. Conclusions

Bee products constitute an important component of medicines and cosmetics. Honey is regenerative and antimicrobial due to its high osmolarity, the presence of hydrogen peroxide and lysozyme. Manuka honey thanks to the presence of methylglyoxal is a potent antiseptic agent. Propolis is a bee product rich in phenolic compounds, which determine antimicrobial, UV protective, analgesic, antioxidative and regenerative activities. Royal jelly is characterized by the presence of royalisin and jelleines peptides. It also contains 10-hydroxy-*trans*-2-decenoic acid which improves the production of collagen and is antiseptic. Bee pollen is rich in unsaturated fatty acids, vitamins, flavonoids and hydroxy acids. Beeswax plays the most important role as emulsifier of the cosmetic forms. Moreover, bee venom is an attractive and effective natural toxin rich in peptides. It plays an important role in treatment and care skin especially in photodamage, acne, atopic dermatitis, alopecia or psoriasis. Bee venom exhibits anti-inflammatory, antimicrobial, antifungal and antiviral action. Each of the bee products is characterized by the content of certain active substances, which differentiates one bee product from another, and causes that each of them is worth using for a different skin problem. The effect of bee products on the skin has been proved by numerous studies, whose results are satisfactory, and the use of these product in wound healing highlights their curative value. The advantage of medicines and cosmetics based on bee products is their effectiveness with minimal side effects. Table 2 summarizes skin diseases where the therapeutic application of bee products has been studied.

## Figures and Tables

**Table 1:** Main effects of selected flavonoids and phenolic acids on skin.

**Group**	**Representative**	**Structure**	**Effect**
Flavones	Chrysin	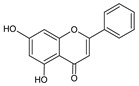	anti-inflammatory [73], antibacterial & antiviral [74], antioxidant [22]
	Apigenin	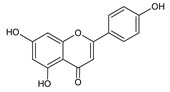	antiviral & antifungal [74], anti-allergic [75], antioxidant [22]
Flavonols	Galangin	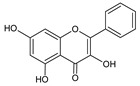	antiviral [62], antifungal [76], antioxidant [22]
	Kaempferol	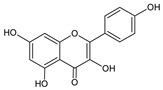	anti-inflammatory [77], antifungal & antiviral [74], antioxidant [22], UV photoprotective [78]
	Quercetin	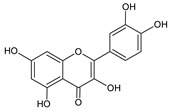	anti-allergic [2], antiviral & antifungal [74], antibacterial [12], antioxidant [79], UV photoprotective [78], anti-inflammatory [77]
Flavanones	Pinocembrin	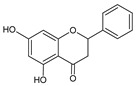	antifungal [76], antioxidant [22]
	Naringenin	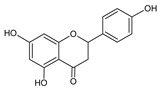	UV photoprotective [80], antioxidant [79], anti-inflammatory [77], antiviral [74]
Phenolic acids	*p*-Coumaric	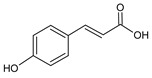	antiviral [20], antibacterial [46]
	Caffeic	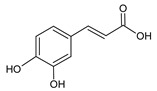	anti-inflammatory [55], antiviral [20], antibacterial [46], antifungal [59]
	Ferulic	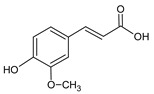	antibacterial [46], photoprotective [60]

**Table 2:** The summary of the skin diseases where the therapeutic application of bee products has been studied.

Bee Product	Components	Effect	Disease	Reference
**Honey**	pinocembrin, lysozyme	antifungal	tinea	[13,46,64]
	methylglyoxal, defensin-1 peptide, lysozyme, glucose oxidase, phenolic acids	antibacterial	wounds, burns, ulcers	[13,16,45,46,50]
	fruits acids, sugars	exfoliating	wrinkles	[13]
	quercetin, naryngenin, kaempferol, chrysin	anti-inflammatory	wounds, gum inflammation	[49,52]
	carbohydrates, fruit acids, trace elements	regenerative	wounds	[40,49,52,53]
**Propolis**	chrysin	analgesic	wounds	[3]
	caffeic acid, quercetin	anti-inflammatory	wounds	[19,55]
	pinocembrin, galangin, caffeic acid	antibacterial	acne, wounds	[19,57,65,66]
	pinocembrin, pinobanksin, quercetin, kaempherol, caffeic acid, *p*-coumaric acid, terpenes,	antifungal	tinea, fungal infections	[19,59]
	galangin, kaempferol, quercetin	antiviral	infection of *Herpes simplex* virus	[62,63]
	caffeic acid, *p*-coumaric acid, ferulic acid, quercetin, kaempferol	photoprotective	photoaging	[60]
	phenolic acids, flavonoids	antiaging	wrinkles	[59]
	genistein	stimulates angiogenesis	diabetic wound	[56]
**Royal jelly**	defensin-1 peptid, ferulic acid	antibacterial	wounds, diabetic foot ulcers, acne	[32,67,68]
	10-hydroxydecanoic acid, 3-10-dihydroxydecanoic acid, amino, gamma globulin	antiinflammatory	atopic dermatitis, wounds, hypertrophy, hyperkeratosis	[67]
	10-hydroxy-*trans*-2-decenoic acid, 10-hydroxydecanoic acid	antiaging	wrinkles	[67]
	10-hydroxydecanoic acid, 3-10-dihydroxydecanoic acid	immunomodulatory and antiallergenic	autoimmune and inflammatory diseases	[70]
**Bee pollen**	pinocembrin, apigenin, quercetin, kaempferol, ferulic acid, *p*-coumaric acid	antifungal	tinea	[2,24,43]
	kaempferol, phenolic acids	antimicrobial	burns	[2,6,24,43]
	phenolic acids, fatty acids, phytosterols, kaempferol, quercetin	antiinflammatory	atopic dermatitis, burns	[2,43]
	methionine, zinc, phospholipids	sebo-balancing	acne	[43]
**Beeswax**	squalene, 10-hydroxy-*trans*-2-decenoic acid, chrysin	antibacterial	wounds, atopic dermatitis, psoriasis	[34,36]
	sterols	reduce transepidermal water loss	atopic dermatitis	[34]
**Bee venom**	melittin	antimicrobial	wounds, acne	[39,81,82,83,92]
	melittin, apamin	antifungal	tinea	[84,85,86]
	melittin	antiviral	herpes simplex infections	[84,85,86,87]
	not reported	photoprotective, antimelanogenic	hiperpigmentation	[44,94]
	melittin, adolapin	antiinflammatory	plaque psoriasis, wounds, atopic dermatitis	[83,84,89,91,92]
	phospholipase A2	pigmentation effect	vitiligo	[39]
	not reported	promote hair growth	alopecia	[88]
	not reported	antiwrinkle	wrinkles	[44,94]
